# Prehabilitation in Modern Colorectal Cancer Surgery: A Comprehensive Review

**DOI:** 10.3390/cancers14205017

**Published:** 2022-10-13

**Authors:** Augustinas Bausys, Marius Kryzauskas, Vilius Abeciunas, Austeja Elzbieta Degutyte, Rimantas Bausys, Kestutis Strupas, Tomas Poskus

**Affiliations:** 1 Clinic of Gastroenterology, Nephrourology and Surgery, Institute of Clinical Medicine, Faculty of Medicine, Vilnius University, 03101 Vilnius, Lithuania; 2 Department of Abdominal Surgery and Oncology, National Cancer Institute, 08660 Vilnius, Lithuania; 3 Center for Visceral Medicine and Translational Research, Institute of Clinical Medicine, Faculty of Medicine, Vilnius University, 03101 Vilnius, Lithuania; 4 Faculty of Medicine, Vilnius University, 03101 Vilnius, Lithuania

**Keywords:** colon cancer, rectal cancer, colorectal cancer, prehabilitation, exercise, surgical outcomes

## Abstract

**Simple Summary:**

Surgical resection is the primary curative treatment option for colorectal cancer. However, colorectal resections remain associated with significant postoperative morbidity and mortality. Furthermore, most rectal cancer patients and some patients with locally advanced colon cancer may need preoperative neoadjuvant therapy. It improves long-term outcomes but impairs patients’ physical fitness and thus further increases surgical risk. Prehabilitation is a novel approach, aiming to improve patients’ physical and psychological capacity to reduce postoperative morbidity and improve treatment outcomes. This study aims to comprehensively overview current knowledge on colorectal cancer surgery’s prehabilitation.

**Abstract:**

Colorectal cancer remains the third most prevalent cancer worldwide, exceeding 1.9 million new cases annually. Surgery continues to be the gold standard treatment option. Unfortunately, colorectal cancer surgery carries significant postoperative morbidity and mortality. Moreover, most rectal cancer patients and some patients with locally advanced colon cancer require preoperative neoadjuvant therapy. It improves long-term outcomes but impairs patients’ physical fitness and thus further increases surgical risk. Recently, prehabilitation has gained interest as a novel strategy to reduce treatment-related morbidity for patients undergoing colorectal cancer surgery. However, the concept is still in its infancy, and the role of prehabilitation remains controversial. In this comprehensive review, we sum up present evidence on prehabilitation before colorectal cancer surgery. Available studies are very heterogenous in interventions and investigated outcomes. Nonetheless, all trials show at least some positive effects of prehabilitation on patients’ physical, nutritional, or psychological status or even reduced postoperative morbidity. Unfortunately, the optimal prehabilitation program remains undetermined; therefore, this concept cannot be widely implemented. Future studies investigating optimal prehabilitation regimens for patients undergoing surgery for colorectal cancer are necessary.

## 1. Introduction

Colorectal cancer (CRC) remains the third most prevalent cancer worldwide, exceeding 1.9 million new cases annually [1]. Surgery continues to be the gold standard treatment option. However, colorectal cancer surgery carries significant postoperative morbidity and mortality [2]. Moreover, the standard advanced rectal cancer management regimen includes neoadjuvant chemoradiotherapy (CRT) with or without neoadjuvant chemotherapy [3,4,5]. Similarly, some locally advanced colon cancer cases may also benefit from neoadjuvant chemotherapy [6]. Despite neoadjuvant cytotoxic therapy improving long-term oncological outcomes, it significantly decreases physiological reserve by deteriorating patients’ physical condition and nutritional status and promoting sarcopenia [7]. Thus, it may be very challenging for patients. Despite the modern CRC treatment achieving good oncological outcomes, the patients are at significant risk of suffering various treatment-related adverse events and complications through the treatment journey [2].

Physical fitness has been linked not only with a lower risk of developing CRC but also with enhanced recovery and better treatment outcomes in the case of the disease [8,9,10,11,12]. Further exercise interventions are considered to be safe and feasible in oncologic patients, including CRC patients before the surgery and afterward, at the time of adjuvant chemotherapy. Additionally, preoperative exercise-based interventions have positive impact on various health-related outcomes, including improved physical fitness [13,14,15]. Therefore, prehabilitation has gained interest as a novel strategy to reduce treatment-related morbidity [16,17,18,19]. The definition of prehabilitation is not yet standardized. Currently, it may be defined as any interventions initiated preoperatively, aiming to strengthen patients’ physical, nutritional, medical, and mental condition to increase patients’ capacity for resisting surgical trauma and facilitating postoperative return to preoperative conditions [20]. However, this is a relatively new concept and still an investigational treatment option. Present studies on prehabilitation in CRC patients are very heterogenous in studied interventions and outcomes. Thus, the role of prehabilitation in modern CRC treatment remains controversial. This comprehensive review summarizes today’s evidence on prehabilitation’s role in modern CRC surgery.

## 2. Materials and Methods

**Figure 1:**
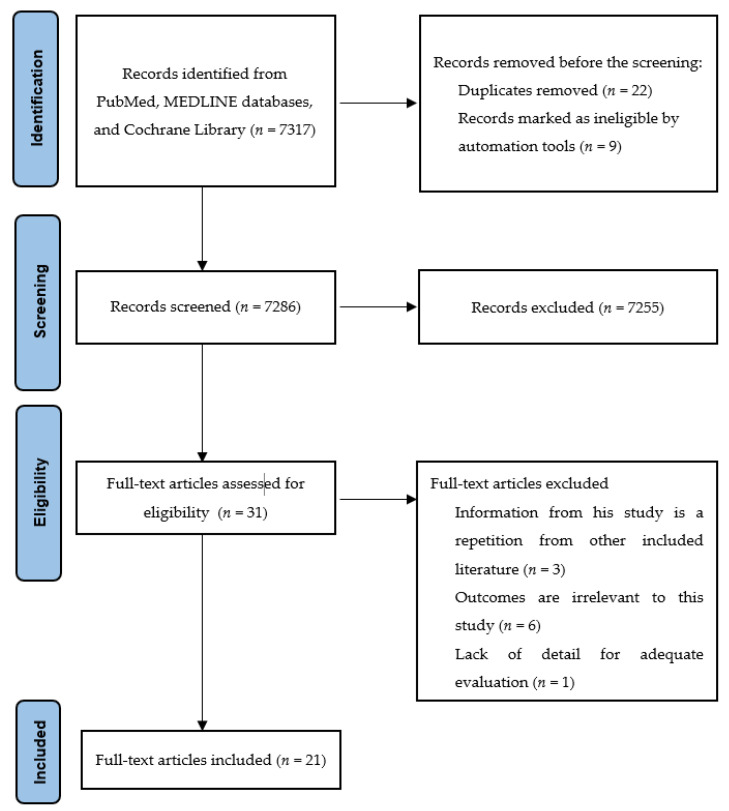
Literature search flow diagram.

An extensive literature search was performed utilizing the PubMed, MEDLINE databases, and Cochrane Central Register of Controlled Trials on 1 May 2022. The following Medical Subject Heading (MeSH) terms were used during the search process: (prehabilitation OR exercise OR physical therapy OR physical fitness OR nutritional support OR psychological support) AND (colon cancer OR rectal cancer OR colorectal cancer). Only English language manuscripts were considered. As a first step, articles were included based on their title. Next, two independent experienced reviewers (V.A. and A.E.D.) reviewed the titles and identified the appropriate abstracts. After all original studies investigating prehabilitation for CRC were identified and included in this comprehensive review. Full-text manuscripts were extracted for confirmation of inclusion (Figure 1). Jadad [21] and the Newcastle–Ottawa [20] scales for randomized and non-randomized studies were used to determine each study’s quality of evidence. Institutional review board approval was not required.

## 3. The Present Concept of Prehabilitation in Surgical Management of Colorectal Cancer 

The definition of prehabilitation is not yet standardized. Currently, it may be defined as any interventions initiated preoperatively, aiming to strengthen patients’ physical, nutritional, medical, and mental condition to increase patients’ capacity for resisting surgical trauma and facilitating a postoperative return to preoperative conditions [16]. There is a clear emphasis on the time-sensitive component [22]. The preoperative period provides a unique window to condition patients for the upcoming physiological and psychological stress, because most are willing to modify behavior for improved outcomes [23,24]. Today, prehabilitation remains an experimental treatment modality, and there is no general agreement on the optimal design of such programs. Available protocols may include one (unimodal) or several (multimodal) interventions to improve patients’ physical fitness and capacity, optimize nutritional status, and promote psychological resilience. The real benefits of prehabilitation also remain the topic for discussion because today’s evidence is very contradictory. Some studies report minimal benefits regarding the decreased length of hospital stay (LOS) [25]. At the same time, others show larger-scale benefits such as improved nutritional status and physical performance as well as better quality of life (QoL) or even up to 50% lower postoperative morbidity [23,26,27,28]. The variety of interventions, differences in measured outcomes, and heterogeneity of results challenge standardization and wide adoption of this approach. The different prehabilitation programs investigated and their interventions are summarized in Table 1. 

Despite all studies investigating prehabilitation for CRC surgery, they were very different in interventions, timing, and measured outcomes. Table 2 provides more details on study design and measured outcomes as well as each study’s quality of evidence.

Among 20 available studies on prehabilitation for CRC patients, there are 10 randomized controlled trials (RCTs) [26,27,30,31,32,37,38,39,40,43], 9 pilot studies [16,17,29,33,34,35,36,41,44] and 1 retrospective cohort study [42]. Table 3 summarizes the reported outcomes of selected studies.

### 3.1. Exercise Programs Used in Unimodal and Multimodal Prehabilitation

It is well known that exercise in the perioperative period is safe and has many benefits for patients’ health. Exercise has been shown to improve physical fitness, enhance the quality of life, alleviate depression and anxiety symptoms, and reduce cancer-related fatigue [17,29,34]. Thus, it is unsurprising that most studies on prehabilitation in CRC patients investigated unimodal exercise-based programs [16,26,29,30,31,32,33,34,35,36,43,44]. Today, there is no consensus on the best exercise program for CRC patients. This fact explains the heterogeneity of interventions throughout the available studies. Different interventions may include aerobic, resistance, and other training options or combinations. These different types of exercise have various benefits for human health. Even a short intervention with aerobic training (2–3 weeks) was shown to elicit improvements in physical fitness, cardiac, respiratory, and musculoskeletal function [10]. Resistance training is known to stimulate muscle hypertrophy and increase muscle mass, strength, and function. Crucially, it is effective in any age group, including frail elderly patients, who have the highest risk for postoperative complications following CRC surgery [45].

Studies included in this review showed that unimodal exercise prehabilitation consisting of aerobic and/or resistance exercises is a safe and viable option for CRC patients [29,30,31,33,34,35,40,41,43]. Additionally, it positively impacts fitness level (improved VO_2peak_, 6-minute walking distance scores, functional walking capacity), leg (e.g., quadriceps), arm, and inspiratory muscle strength. Different tools and outcomes were used to objectify exercise’s impact on a patient’s physical condition. Five studies [16,26,29,30,41,44] measured VO_2peak_ and VO_2_ at the ventilatory anaerobic threshold (VAT). All, except one [41], of these studies showed improved VO_2_ after prehabilitation. Three studies by Moug and Loughney [31,32,36] investigated the effects of prehabilitation on daily step count and showed a positive impact on the parameter. Previous knowledge indicates that lower physical activity levels, determined by daily step count, are associated with increased rehospitalization rates and poor adherence to neoadjuvant treatment protocols [46]. Thus, prehabilitation may be considered to have the potency to improve postoperative outcomes and patients’ ability to tolerate neoadjuvant treatment [40,42,46]. Another common parameter investigated in a series of studies [17,31,34,35,38,40,42] is the 6-minute walking test (6 MWT) results. All available studies except one [40] show that prehabilitation improves 6 MWT outcomes. This improvement of objective functional reserves representing parameters indicates that prehabilitation improves CRC patients’ physical condition before surgical trauma and that intervention may have therapeutic benefits [26,27,42]. Moreover, seven studies [26,32,33,34,35,41,42] presented the prehabilitation effect on different skeletal muscle functions representing parameters. Every trial showed at least a slight improvement in muscle strength and endurance after prehabilitation. Such impact is relevant because lower muscle mass is associated with impaired postoperative outcomes in cancer patients [9]. Additionally, one study [43] showed that prehabilitation also improves inspiratory muscle strength, and this improvement was linked with a minor decrease in postoperative hospital stay and improved recovery. Besides positively impacting physical capacity, exercise interventions enhanced the quality of life (reduced depression and anxiety symptoms scores) [16,17,26,27,29,30,31,32,33,34,35,36,38,41,42,43,44]. The exercise interventions were effective in both unimodal and multimodal prehabilitation settings.

### 3.2. Nutritional and Psychological Interventions Used in Multimodal Prehabilitation

Malnutrition is the most common comorbidity in cancer patients [47], affecting 30% to 60% of patients with CRC [48]. This is mainly due to systemic inflammation caused by cancer cells. Cancer expansion triggers the release of proinflammatory cytokines such as IL-1, IL-6, and TNF-α, which in turn increase lipolysis, muscle breakdown, and insulin resistance. All these effects lead to muscle wasting with or without loss of adipose tissue [49]. The oncological patients frequently have impaired physical status and decreased quality of life, preventing adherence to therapy, reducing efficacy and tolerability, and worsening the prognosis. Cancer cachexia remains a decisive, independent prognostic factor for poor treatment outcomes [50,51]. Timely nutritional intervention can improve prognosis as well as decrease rates of morbidity and mortality among cancer patients [52]. Thus, dietary interventions appear to be a good part of multimodal prehabilitation programs in CRC management [17,27,37,39,40,41,42].

Currently, eight studies [17,27,37,38,39,40,41,42] investigated the effect of different nutritional interventions. They included personalized dietary counseling, whey protein supplementation, or a complete diet by providing daily meals. All but one [39] trial found at least some beneficial effect of nutritional support resulting in increased muscle mass and reduced fat mass. These studies indicate that multimodal prehabilitation, which includes nutritional support, could be superior to unimodal prehabilitation in terms of functional status improvement. However, randomized trials are necessary to confirm this hypothesis.

In addition to all the physiological challenges affecting CRC patients, psychological and emotional distress cannot be forgotten. The prevalence of depression among cancer survivors can be as high as 49% [53]. Even for patients with no history of psychological disorders, the extreme burden of cancer diagnosis increases the risk of mental disorders, which can harm patients’ adherence to treatment, postoperative recovery, and quality of life [54]. Personal risk factors for depression include such demographic factors as gender, age, and socioeconomic factors - unemployment and lack of social support [55,56,57]. A biological explanation for increased psychological stress in cancer patients includes a hyperactive hypothalamic-pituitary-adrenal axis, glutamate excitotoxicity, and inflammation [58]. Because psychological stress is a modifiable risk factor for poor CRC treatment outcomes, there is a rationale to address it via specific prehabilitation. 

Four studies in CRC patients included psychological support as a part of multimodal prehabilitation [17,27,37,40]. All of them incorporated breathing and relaxation exercises as anxiety-reducing techniques. However, only one trial [17] measured psychological well-being as an outcome. This trial determined that prehabilitation reduced symptoms of anxiety and depression before surgery. However, it had no impact on any domain of health-related quality of life (HRQOL). It is challenging to evaluate the effect of psychological prehabilitation because current studies lack appropriate outcomes. 

## 4. Discussion

### 4.1. Considerations for the Wider Use of Prehabilitation Programs in Colorectal Cancer Surgery Patients and Existing Gaps in Prehabilitation Research 

This review provided an overview of available evidence of prehabilitation use in the management of CRC. Current trials are very heterogeneous in design, used interventions, and evaluated outcomes. Nonetheless, all trials show at least some positive effects of prehabilitation on patients’ physical, nutritional, or psychological status or even reduced postoperative morbidity [16,17,26,27,29,30,31,32,33,34,35,38,42,43,44]. The differences in available trials preclude the broad implementation of prehabilitation in CRC management despite a sufficient amount of evidence encouraging the use. Physicians seeking to implement prehabilitation in CRC management will have a number of questions, with some having no answer because of the gaps in present knowledge.

#### 4.1.1. Question 1: Which Prehabilitation Should Be Used: Multimodal or Unimodal?

The best modality of prehabilitation remains unknown. Both unimodal and multimodal prehabilitation programs can be used for CRC management [59], with comparable effects on clinical outcomes, fitness, and quality of life. Considering that CRC patients suffer physical, nutritional, and psychological burdens [60], it appears that multimodal prehabilitation may be superior [61]. The downside of multimodal prehabilitation mainly lies in the additional resources (both financial and human) needed for adequate care. Ongoing clinical trials investigating multimodal prehabilitation in CRC patients will shed more light on this topic [61].

#### 4.1.2. Question 2: Is Supervised Prehabilitation Superior to the Home-Based Programs?

Prehabilitation is usually implemented either supervised by a medical professional in a health care facility or, after introductory training, in-home setting. Each modality carries its respective advantages and disadvantages. Supervised prehabilitation allows for the monitoring of adherence and swift implementation of any necessary changes. Supervised exercises in patients with chronic low back pain [62], intermittent claudication [63], or after anterior cruciate ligament reconstruction [64] were shown to improve the outcomes. However, the supervised prehabilitations protocols carries significant logistical challenges for patients and healthcare providers. During the COVID-19 pandemic, a new way to provide supervised prehabilitation appeared. This can be achieved via tele-prehabilitation, where patients are supervised utilizing video conferencing applications. This eliminates the logistical challenges of supervised prehabilitation while maintaining all the advantages. Current studies indicate that patients prefer home-based prehabilitation; thus, a higher level of adherence could be achieved [27]. Prehabilitation in a home setting with or without telemonitoring may seem the most effective and rational approach for most patients with CRC. On the other hand, there is a great discussion regarding this topic in the current literature. Most scepsis and criticism for the home-based approach are given for unclear safety and efficacy of this approach. Additionally, the rates of non-compliance and attrition for facility-based prehabilitation may be overestimated [65,66,67,68]. Thus, there is a place for studies that would directly compare home-based vs. supervised prehabilitation for CRC cancer patients.

#### 4.1.3. Question 3: How to Make Sure Patients Comply with Prehabilitation?

Poor compliance remains one of the significant obstacles in current prehabilitation regimens and results in worse-than-expected outcomes [69]. Therefore, finding ways to increase compliance remains the most important. Direct supervision by healthcare professionals could boost patients’ motivation and readiness to adhere to the prehabilitation regimen [70]. Although, as highlighted before, hospital-based prehabilitation has some significant logistical challenges. These challenges could be overcome by a hybrid approach or switching to tele-prehabilitation. In addition, psychological support could be implemented as it may improve motivation for adherence [71]. In our review, only four studies included some psychological prehabilitation [17,27,37,40]. Further research in this field is necessary to delineate ways to ensure maximal adherence. 

#### 4.1.4. Question 4: When Should the Prehabilitation Be Started?

As the time window between diagnosis and surgery in CRC patients is relatively short, the prehabilitation in patients undergoing only surgery should begin without delay. For patients receiving neoadjuvant treatment, several options are available. One possibility would be the window between the end of neoadjuvant therapy and surgery, which should ideally last 3–5 weeks [72]. Many studies in this review, however, utilized prehabilitation in conjunction with neoadjuvant CRT, where the feasibility of prehabilitation has already been established [31]. Prehabilitation has been proven to alleviate some negative impacts of neoadjuvant therapy, including declining physical fitness, increased depression and anxiety rates, and reduced HRQOL [10]. Therefore, prehabilitation should be started without delay to minimize the significant declines in function from NACRT and improve surgical outcomes.

#### 4.1.5. Question 5: What Benefits Could Prehabilitation Bring to CRC Patients?

Current evidence on prehabilitation’s impact on postoperative outcomes is controversial. Five studies investigating prehabilitation’s impact on postoperative morbidity showed a positive effect [11,26,27,30,42]. Similarly, only 2 of 6 studies that investigated prehabilitation’s impact on the length of hospital stay showed a positive effect [17,27,38,40,42,43]. Additionally, 1 study demonstrated that prehabilitation promoted neoadjuvant therapy-induced tumor regression [30], but these findings were not confirmed in another study [44]. Taken together, it is likely that prehabilitation has a positive effect on clinical outcomes in CRC patients. Still, currently, there is a lack of studies designed to confirm this. 

### 4.2. Limitations of the Current Knowledge

There are many limitations in the present comprehensive review and current knowledge on prehabilitation for CRC surgery. First, this study is a comprehensive, but not a systematic review. Second, the analysis is limited by significant heterogeneity of available studies in terms of patients and oncologic treatment pathways (upfront surgery vs. surgery after neoadjuvant treatment), different interventions (unimodal exercise prehabilitation vs. multimodal prehabilitation), and measured outcomes. Such limitation rises from the lack of prehabilitation standardization and consequently the nature of the current literature on this topic. Third, there is only limited data from large-scale randomized studies. Therefore, current knowledge on prehabilitation for CRC has to be addressed with caution, and further studies are needed to elucidate remaining unclarities which were highlighted through this review.

## 5. Conclusions

Prehabilitation is a new approach to improve patients’ physical, and nutritional status and psychological well-being before surgery. This comprehensive review summarized the currently available data on the prehabilitation in the management of CRC. Even though the majority of studies were not homogenous in their design and interventions, the majority showed at least some benefits: improved physical performance and nutritional status, reduced length of hospital stay and postoperative complication rate, as well as improved quality of life. However, more research on optimal prehabilitation techniques is needed to establish the best prehabilitation strategy for managing colorectal cancer patients.

## Figures and Tables

**Table 1:** Details on interventions in colorectal cancer surgery prehabilitation programs.

Author; Year	Prehabilitation Group			Control Group
	Type of Intervention (Unimodal vs. Multimodal)	Timing	Interventions Used for Prehabilitation	
Berkel et al. [26]; 2022	Unimodal	Start three weeks before surgery.	Exercise intervention: personalized supervised exercise program consisting of 60-mintraining sessions combining 40 min of moderate-to-high intensity interval training on a cycle ergometer to improve aerobic fitness and 20 min of resistance training to improve peripheral muscle strength.	Standard of care
Alejo et al. [29]; 2019	Unimodal	Start during five weeks of CRT and continuation for additional 6–8 weeks before surgery.	Exercise intervention: 6 practical classes on aerobic (running and walking at RPE = 6–10), resistance (exercises for biceps, triceps, chest, abdominal, lumbar, quadriceps, iliotibial tract, and calves), and outdoor flexibility exercises conducted in a park.	N/A
Morielli et al. [30]; 2021	Unimodal	Start during neoadjuvant CRT.	Exercise intervention: personalized HIIT exercise program on a treadmill. Each HIIT session consisted of a 2-min, high-intensity interval completed at 85% of VO_2_ peak followed by 2 min of active recovery completed at 40% of VO_2_ peak. The number of HIIT intervals started with five and progressed by one every other session until participants reached the maximum number of 8 intervals.	Standard of care
West et al. [16]; 2015	Unimodal	Start 6 weeks before surgery during neoadjuvant CRT.	Exercise intervention: in-hospital supervised exercise training program consisting of 40 min interval training on a cycle ergometer. The exercise intensity was modified based on individual ramped CPET protocol results.	Standard of care
Moug et al. [31]; 2019	Unimodal	Start before neoadjuvant CRT; a minimum of 13 weeks duration: 5 weeks during neoadjuvant CRT followed by a minimum of 8 weeks of exercises before surgery.	Exercise intervention: initial exercise counseling session was followed by a 13–17-week telephone-guided walking program. The program was targeted at counting steps: during the first eight weeks, the step count goal was gradually increased from the baseline and then maintained or increased over the remaining weeks. The target was to increase the average daily step count by 3000 over the baseline by week 8.	Standard of care
Moug et al. [32]; 2020	Unimodal	Start before neoadjuvant CRT; a minimum of 13 weeks duration: 5 weeks during neoadjuvant CRT followed by a minimum of 8 weeks of exercises before surgery.	Exercise intervention: initial exercise counseling session was followed by a 13–17-week telephone-guided walking program. The program was targeted at counting steps: during the first eight weeks, the step count goal was gradually increased from the baseline and then maintained or increased over the remaining weeks. The target was to increase the average daily step count by 3000 over the baseline by week 8.	Standard of care
Singh et al. [33]; 2017	Unimodal	Start over a period of 16 weeks before surgery.	Exercise intervention: supervised exercise program consisting of two 60-minute aerobic and resistance exercise sessions per week. Resistance exercises aimed at training the major muscle groups included seated row, chest press, latissimus dorsi pull-down, leg extension, curl, and press exercises. The aerobic exercise included walking or jogging, or a treadmill, or cycling, or rowing.	N/A
Singh et al. [34]; 2018	Unimodal	Start over a period of 10 weeks during neoadjuvant CRT.	Exercise intervention: supervised program consisting of aerobic and resistance exercises. Resistance exercises aimed at training the major muscle groups included seated row, chest press, latissimus dorsi pull-down, leg extension, curl, and press exercises. The aerobic exercise included walking or jogging, or a treadmill, or cycling, or rowing.	N/A
Heldens et al. [35]; 2016	Unimodal	Start during neoadjuvant CRT, and the exact duration depending on the individual decision for surgery timing.	Exercise intervention: supervised individualized endurance and resistance exercise program. The endurance program utilized treadmill and cycling exercises. Chest and leg press, along with the lateral pull down at 40% 1-RM, were used in the resistance exercise program.	N/A
Loughney et al. [36]; 2017	Unimodal	Start after completion of neoadjuvant CRT; 6-week duration.	Exercise intervention: supervised in-hospital exercise training program. Training intensity was determined individually by the results of CPET at weeks 0 and 3. Exercise training included 40 min of interval training on an electromagnetically braked cycle ergometer with alternating intensity.	Standard of care
Gillis et al. [37]; 2019	Multimodal	Started 4 weeks before surgery; continued 8 weeks after surgery.	Exercise intervention: both groups carried out a home-based exercise program with one group additionally employing supervised group exercise sessions once per week Nutrition intervention: individualized counseling on diet and supplementation with whey protein. Additionally, the individualized nutrition care plans focused on alleviating cancer-related symptoms, blood glucose control, optimization of body composition, and optimizing nutrient intake guided by actual intake of those nutrients. Anxiety-reducing intervention: deep breathing exercises along with individualized relaxation exercises.	Patients receiving rehabilitation
Gillis et al. [38]; 2016	Unimodal	Start 4 weeks before surgery; continued for 4 weeks after surgery.	Nutrition intervention: 90 min. Counseling sessions by a registered dietitian to assess nutritional status; individualized nutrition care plan with daily supplements guided by analysis of food consumption and estimated daily needs. An appropriate quantity of whey protein supplement was used to compensate for the estimated deficit in daily protein consumption.	Individualized nutrition counseling with a non-nutritive placebo
Furyk et al. [39]; 2021	Multimodal	Start 4 weeks before surgery.	Exercise intervention: personalized supervised program consisting of three 1-hour sessions of aerobic and resistance exercises each week performed on non-consecutive days. The sessions included 30 min strength and core/balance circuit followed by 20 min of aerobic exercise. Nutrition intervention: Personalized dietary counseling in line with Australian Dietary Guidelines.	Standard of care
Bousquet-Dion et al. [40]; 2018	Multimodal	Start 4 weeks before surgery.	Exercise intervention: home-based exercise program with an additional supervised workout session. Nutrition intervention: personalized dietary counseling with daily whey protein supplementation. Additionally, the customized nutrition care plans focused on alleviating cancer-related symptoms, blood glucose control, optimization of body composition, and optimizing nutrient intake guided by actual intake of those nutrients. Anxiety-reducing intervention: deep breathing instructions and personalized relaxation exercises.	Standard of care
Tweed et al. [41]; 2021	Multimodal	Start 4 weeks before surgery.	Exercise intervention: Personalized, supervised strength and aerobic training program. For the strength training, six functional upper and lower body push-pull exercises were used (shoulder press, chest press, lateral pull down, deadlift, leg press, seated row). Aerobic training consisted of HIIT on the cycle ergometer; an exercise intensity was guided by personal ventilatory thresholds measured with CPET. Nutrition intervention: three freshly prepared high-protein meals and three snacks per day. The nutrients contained the required amount of protein and calories as calculated by the dietitian. Patients were not permitted to eat other foods.	N/A
Klerk et al. [42]; 2021	Multimodal	Start at least 4 weeks before surgery; duration was adjusted based on the date of surgery.	Exercise intervention: Personalized, supervised high-intensity training and individual low-intensity training. Nutrition intervention: individualized nutritional advice to meet energy and protein needs. Other interventions: Outpatient clinic consults on smoking and alcohol cessation, preoperative anemia treatment, polypharmacy reduction.	Standard of care
Arias et al. [27]; 2021	Multimodal	Start 30 days before surgery; continued for 30 days after hospital discharge.	Exercise intervention: Combination of aerobic and muscular resistance exercises performed at home for the approximate duration of 30–45 min, guided by a video playlist. Nutrition intervention: Dietary recommendations, nutritional supplementation with protein-rich and high in vitamin D and CaHMB content foods. Relaxation exercises: Breathing and relaxation exercises.	Standard of care
Karlsson et al. [43]; 2019	Unimodal	Start at least 2 weeks before surgery.	Exercise intervention: Inspiratory muscle training performed using the handheld electronic device Power Breathe K3; functional strength workouts performing high-intensity exercises (such as chair stands and step-ups with weight belts) and endurance training (such as interval walking indoors and/or outdoors, bouts of stair climbing, and Nordic walking outdoors).	Standard of care
West et al. [44]; 2019	Unimodal	Start 6 weeks before surgery.	Exercise intervention: Tailored exercise program consisting of 40 min interval training using an electromagnetically braked cycle ergometer.	Standard of care
Li et al. [17]; 2013	Multimodal	The start date was predetermined by the time remaining until surgery alone.	Exercise intervention: Aerobic exercise sessions (30 min of walking or using an aerobic exercise machine) combined with resistance training (calisthenics and elastic band movements). Nutrition intervention: Excess alcohol or fat intake reduction counseling; whey protein isolate provided to guarantee a daily intake of protein. Anxiety-reducing intervention: relaxation and breathing exercises.	Standard of care

N/A—not applicable; CRT—chemoradiotherapy; HIIT—high-intensity interval training; CaHMB—calcium-β-hydroxy-β-methylbutyrate.

**Table 2:** Characteristics of prehabilitation studies of surgical management for colorectal cancer.

Author Year	Design	Description and Number of Participants (*n*)	Measured Outcomes	N-O Score	Jadad Score
Berkel et al. [26]; 2022	RCT	Colorectal cancer patients undergoing colorectal resection (*n* = 57)	Primary outcome: 30-day postoperative complication rate as determined by the Clavien–Dindo classification Secondary outcomes: Changes in preoperative aerobic fitness level, determined by VO_2_ at the VAT LOS Muscle strength, determined by handgrip strength and quadriceps strength Unplanned readmissions 30 and 90 days after surgery	N/A	3
Alejo et al. [29]; 2019	Non-randomized pilot study	Colorectal cancer patients undergoing neoadjuvant treatment (*n* = 12)	Primary outcome: Adherence to the intervention Secondary outcomes: Quality of life, determined by EORTC QoL questionnaire C-30 Anxiety and depression scores, determined utilizing the Hospital Anxiety and Depression Scale BMI Fitness level, using a one-mile walk for evaluation of cardiorespiratory fitness, as well as handgrip strength using a dynamometer, and a 5-repetition sit-to-stand test on a straight-backed chair Physical activity, assessed via spontaneous PA levels through accelerometry	7	N/A
Morielli et al. [30]; 2021	RCT	Rectal cancer patients to be treated with neoadjuvant CRT (*n* = 36)	Primary outcome: Cardiorespiratory fitness level, determined by VO_2_ peak while performing modified Bruce protocol stress test with direct gas exchange and ventilation measurements Secondary outcomes: Functional fitness, determined by Senior’s Fitness Test	N/A	3
West et al. [16]; 2015	Non-randomized, blinded pilot study	Rectal cancer patients to be treated with neoadjuvant CRT (*n* = 39)	Primary outcome: Oxygen uptake at lactate threshold, measured during CPET or bicycle exercise Secondary outcomes: Physical fitness, measured using PA monitor and averaged step count Exercise program’s safety and feasibility for high-risk patients	8	N/A
Moug et al. [31]; 2019	RCT	Rectal cancer patients to be treated with neoadjuvant CRT (*n* = 48)	Primary outcome: Feasibility and acceptability of the research procedures, determined by eligibility and recruitment rates Participant acceptability of randomization (percentage of participants attending baseline measurements after informed consent to participate) Participant retention rate and compliance with the physical activity intervention Secondary outcomes: Median step count per day Height Weight Hip and waist circumference Sit-to-stand test 6 MWT	N/A	3
Moug et al. [32]; 2020	RCT	Rectal cancer patients to be treated with neoadjuvant CRT (*n* = 44)	Primary outcome: Muscle mass, using total psoas area (TPA) measurement	N/A	3
Singh et al. [33]; 2017	Non-randomized pilot study	Rectal cancer patients planned for rectal resection (*n* = 12)	Primary outcome: Muscle strength, determined by chest press, seated row, leg press, and leg extension exercises using the 1-RM method Secondary outcomes: Physical performance assessed using usual and fast 6 m walk, 6 m backwards walk, repeated chair rise, stair climb, and the 400 m walk tests Body composition, using LBM, FBM using dual-energy X-ray absorptiometry Fatigue, determined by the validated 30-item short form of the Multidimensional Fatigue Symptom Inventory QoL, determined by EORTC QoL questionnaire C-30	6	N/A
Singh et al. [34]; 2018	Non-randomized pilot study	Rectal cancer patients to be treated with neoadjuvant CRT (*n* = 10)	Primary outcomes: Muscular strength, evaluated by performing chest press, seated row, leg press, and leg extension using the 1-RM method Muscular endurance, determined by the number of maximum repetitions performed at 70% of the pre-exercise 1-RM weight for chest press and leg press Physical performance, assessed using usual and fast 6 m walk, 6 m backwards walk, repeated chair rise, stair climb, and the 400 m walk	6	N/A
Heldens et al. [35]; 2016	Non-randomized pilot study	Rectal cancer patients to be treated with neoadjuvant CRT (*n* = 13)	Primary outcome: Functional exercise capacity using the 6 MWT Secondary outcomes: Muscle strength, measured by leg extension and chest press submaximal multiple-repetition (X-RM) test Perception of fatigue, assessed by multidimensional fatigue index (MFI) Perception of QoL, assessed using the SF-36 questionnaire	6	N/A
Loughney et al. [36]; 2017	Non-randomized pilot study	Rectal cancer patients to be treated with neoadjuvant CRT (*n* = 39)	Primary outcome: PA level, determined by 72 h monitoring using SenseWear biaxial accelerometer	7	N/A
Gillis et al. [37]; 2019	RCT	Colorectal cancer patients planned for colorectal resection (*n* = 139)	Primary outcomes: Change in LBM, measured using a multi-frequency bioelectrical impedance analysis (BIA) Functional exercise capacity using the 6 MWT Intervention compliance, assessed by patient-filled diary	N/A	3
Gillis et al. [38]; 2016	RCT	Colorectal cancer patients planned for colorectal resection (*n* = 48)	Primary outcome: Changes in functional walking capacity measured using 6 MWT Secondary outcomes: Self-reported PA, assessed using the CHAMPS questionnaire Health-related quality of life, assessed using the SF-36 questionnaire	N/A	3
Furyk et al. [39]; 2021	RCT	Frail colorectal cancer patients planned for colorectal resection (*n* = 106)	Primary outcome: Changes in functional walking capacity measured using 6 MWT Secondary outcome: PA level, using accelerometry Health-related QoL, assessed using EQ-5D, short-form 12 and modified Barthel index. Post-surgical complications	N/A	3
Bousquet-Dion et al. [40]; 2018	RCT	Colorectal cancer patients planned for colorectal resection (*n* = 80)	Primary outcomes: Functional exercise capacity measured with 6 MWT Secondary outcomes: Self-reported PA, assessed using the CHAMPS questionnaire	N/A	3
Tweed et al. [41]; 2021	Non-randomized pilot study	Colorectal cancer patients planned for colorectal resection (*n* = 9)	Primary outcome: Viability of prehabilitation program defined as ≥80% compliance with the exercise and nutritional program Secondary outcomes: organizational viability acceptability of the prehabilitation program, assessed using a questionnaire functional capacity after prehabilitation, determined by CPET measures on a calibrated electronically braked cycle ergometer muscle strength, determined by handgrip strength LOS Postoperative complication rate Readmission rate 30-day and 1-year mortality rates	6	N/A
Klerk et al. [42]; 2021	Retrospective cohort study	Colorectal cancer patients planned for colorectal resection (*n* = 351)	Primary outcome: Postoperative complications rate Secondary outcomes: Unplanned readmission rate LOS Mortality rate	8	N/A
Arias et al. [27]; 2021	RCT	Colorectal cancer patients planned for colorectal resection (*n* = 20)	Primary outcomes: LOS Occurrence of postoperative complications Changes in LBM. determined using the MF-BIA device Changes in FBM, determined using MF-BIA device	N/A	3
Karlsson et al. [43]; 2019	RCT	Colorectal cancer patients planned for colorectal resection (*n* = 23)	Primary outcome: Process feasibility evaluated with the variables recruitment rate, exercise compliance, and acceptability Secondary outcomes: Scientific feasibility including treatment safety, description of dose level and response, and estimation of treatment results	N/A	3
West et al. [44]; 2019	Non-randomized pilot study	Colorectal cancer patients to be treated with neoadjuvant CRT (*n* = 35)	Primary outcomes: Tumor regression, as expressed by ypT, ypTRG Oxygen uptake at lactate threshold	7	N/A
Li et al. [17]; 2013	Non-randomized pilot study	Colorectal cancer patients planned for colorectal resection (*n* = 87)	Primary outcomes: Functional walking capacity, as determined by 6 MWT Secondary outcomes: Complication rate, graded using the Clavien–Dindo classification Self-reported PA, assessed by CHAMPS short form questionnaire Health-related QoL, assessed using the SF-36 questionnaire	8	N/A

RCT—randomized controlled trial; N/A—not applicable; VAT—ventilatory anaerobic threshold; QoL—quality of life; EORTC - European Organization for Research and Treatment of Cancer; BMI—body mass index; CRT—chemoradiotherapy; 6 MWT—6-minute walking test; SF-36—36-Item Short-Form Health Survey; CCI—comprehensive complication index; PA—physical activity; CHAMPS—Community Healthy Activities Model Program for Seniors; CPET—cardiopulmonary exercise testing; LBM—lean body mass; MF-BIA- multi-frequency bioelectrical impedance analysis; FBM—fat body mass; ypTRG—postoperative pathological tumor regression stage; ypT-stage—postoperative tumor stage pathology; 1-RM—1 repetition maximum.

**Table 3:** Reported outcomes of prehabilitation for colorectal cancer surgery.

Author; Year	Impact on Physical Status	Impact on Postoperative Outcomes	Other Effects
Berkel et al. [26]; 2022	Improved VO_2_ at the VAT and VO_2peak_. Quadriceps strength also increased in the prehabilitation group.	Significantly lower complication rate vs. the usual care group.	N/A
Alejo et al. [29]; 2019	Improved VO_2peak_; after the exercise program, a tendency for increased mean levels of moderate to vigorous PA was observed.	N/A	Adherence to the program was 89% (primary outcome). The scores for the depression and the “emotional function” QoL domain were reduced in the prehabilitation group.
Morielli et al. [30]; 2021	Improved VO_2peak_ while VO_2peak_ decreased in the control group.	Prehabilitation increased rates of pCR/near pCR compared to the control group.	No significant differences were observed between groups for grade ¾ toxicities or treatment completion.
West et al. [16]; 2015	Improved VO_2_ at the VAT and VO_2peak_.	N/A	N/A
Moug et al. [31]; 2019	A reduction in step count was observed in both groups, with the prehabilitation group experiencing a lesser decline (non-significant). Prehabilitation increased 6 MWT scores (non-significant).	N/A	The prehabilitation group achieved high levels of satisfaction.
Moug et al. [32]; 2020	A reduction in daily step count was observed in both groups, with a more considerable reduction recorded in the control group. More patients in the intervention group achieved step count improvements at week 12. Prehabilitation increased muscle mass as determined by TPI.	N/A	N/A
Singh et al. [33]; 2017	Prehabilitation increased muscle strength, endurance and preserved lean body mass and ASM.	N/A	No significant changes in any QoL measure or fatigue determined by MFSI scores were reported. There were no significant changes in general well-being at any point in time (assessed using the SF-36 questionnaire) and no adverse effects or health problems related to the exercise program during the training period.
Singh et al. [34]; 2018	Prehabilitation significantly improved muscle strength for the lower limb exercises. While leg press endurance improved, there was no significant change in chest press muscle endurance. Physical performance as measured by 6 m fast walk and 6 m backwards walk improved in the Prehabilitation group. There was no significant change in 400-meter walk time; however, there was a substantial reduction in heart rate immediately after the completion of the test.	N/A	There were significant changes in 3 measures of QoL (emotional function, financial difficulties, diarrhea), with patients also reporting having less constipation. The exercise program did not cause any adverse events.
Heldens et al. [35]; 2016	Prehabilitation increased patient walking distance as determined by 6 MWT and functional exercise capacity (not significant) as well as both leg and arm muscle strength (significantly).	N/A	The feasibility and safety of the program were observed, with a very high attendance rate (95.7%).
Loughney et al. [36]; 2017	Significant improvements in lying down time, sleep efficiency, and duration were reported in the prehabilitation group compared to the control group.In all participants, there was a significant reduction in daily step count, EE, and MET. The apparent improvement in daily step count and overall PAL in the prehabilitation group was not statistically significant compared to the control group.	N/A	N/A
Gillis et al. [37]; 2019	Prehabilitation did not significantly alter body mass compared to rehabilitation. The prehabilitation group had substantially more relative and absolute LBM and less FBM than the control group.	N/A	N/A
Gillis et al. [38]; 2016	The prehabilitation group experienced a clinically meaningful improvement in 6 MWT scores. Recovery rates were similar between groups. No significant differences in self-reported outcomes were observed between the groups.	No significant differences were observed between the groups in an overall 30-day complications rate and severity, emergency department visits and readmission, and median length of stay.	N/A
Furyk et al. [39]; 2021	N/A	N/A	Poor feasibility of an RCT for preoperative prehabilitation in frail colorectal patients was reported.
Bousquet-Dion et al. [40]; 2018	No significant changes in 6 MWD were found between the groups; however, there was a significant correlation between physical activity, energy expenditure, and 6 MWD in the prehabilitation group.	There were no significant differences in the length of stay, emergency department visits, and complications rate between the groups.	Program compliance was 98%.
Tweed et al. [41]; 2021	Prehabilitation improved handgrip strength and exercise capacity. No difference was observed in VO_2max_ and VO_2_ at VAT before and after prehabilitation.	N/A	No adverse effects were reported. Organizational feasibility was achieved. Overall acceptability of interventions was positive.
Klerk et al. [42]; 2021	Prehabilitation improved 6 MWT and 1-RM.	Compared to the standard care group, rehabilitation reduced complication rate, shortened the median stay, and patients had fewer unplanned readmissions. There was no significant difference in mortality between the groups.	N/A
Arias et al. [27]; 2021	Reduced the deterioration of body composition as compared to the control group 45 days after surgery. These differences, however, were attenuated at 90 days.	Prehabilitation reduced hospital stay duration and postoperative complications.	N/A
Karlsson et al. [43]; 2019	Prehabilitation significantly increased inspiratory muscle strength.	No significant increase in complications was observed in the prehabilitation group. The intervention group showed a shorter median length of stay and better recovery, although not statistically significant.	The recruitment rate was low, at only 35%. Compliance was much higher, at 97%. The overall intervention achieved a high level of acceptability.
West et al. [44]; 2019	Prehabilitation reversed the fall in VO_2_ at VAT due to NACRT.	The prehabilitation group had significantly greater ypTRG at the time of surgery, which did not result in a significant difference in the ypT-stage.	N/A
Li et al. [17]; 2013	Postoperative walking capacity improved significantly in the prehabilitation group at weeks 4 and 8. A higher share of patients recovered in the prehabilitation group compared to the standard of care at week 8. In addition, higher levels of physical activity before and after surgery were reported in the intervention group.	Similar postoperative complication rates and length of stay were observed in both groups.	Prehabilitated patients immediately before surgery had significantly decreased anxiety and depression symptoms. No clinically or statistically significant increases in any domains of HRQOL were reported for the prehabilitation group.

VAT—ventilatory anaerobic threshold; N/A—not applicable; PA—physical activity; pCR—pathologic complete response; 6 MWT—6-minute walking test; TPI—total psoas index; MFSI—Multidimensional Fatigue Symptom Inventory; SF-36—36-Item Short Form Health Survey; CCI—comprehensive complication index; EE—energy expenditure; MET—metabolic equivalent; PAL—physical activity level; LBM—lean body mass; FBM—fat body mass; 1-RM—1 repetition maximum; 6MWD—6-minute walking distance; RCT—randomized controlled trial; ypTRG—postoperative pathological tumor regression stage; ypT-stage—postoperative tumor stage pathology; HRQOL—health-related quality of life.
